# Mechanical Behavior of Titanium Alloys at Moderate Strain Rates Characterized by the Punch Test Technique

**DOI:** 10.3390/ma16010416

**Published:** 2023-01-01

**Authors:** Vladimir V. Skripnyak, Kristina V. Iohim, Vladimir A. Skripnyak

**Affiliations:** Department of Mechanics of Deformed Solid Body, National Research Tomsk State University, 634050 Tomsk, Russia

**Keywords:** titanium alloys, dynamic punch test, high strain rates, stress triaxiality

## Abstract

Material characterization at moderate strain rates is an important factor for improving the adequacy and accuracy of analysis of structures operating under extreme conditions. In this paper, the deformation and fracture of Ti-5Al-2.5Sn alloys were studied utilizing the punch test at strain rates up to several hundred per second. Loading velocities from 0.0003 to 15 m/s were realized during the spherical body penetration through a thin titanium plate. To describe the plastic flow and fracture of the Ti-5Al-2.5Sn alloy at strain rates ranging from 0.001 to 10^3^ s^−1^, a micromechanical damage model was coupled with a viscoplastic constitutive model based on the dislocation dynamics. Numerical simulations of the punch test at 15 and 2 m/s were carried out to validate used constitutive relations. It was verified that the simulated fracture shape and deflections were similar to experimental ones. It was found that dynamic punch test is suitable for validation of damage kinetics under complex stress states.

## 1. Introduction

The development of lightweight and reliable structures in various industries, sports, and medicine (as well as efficient technologies for their manufacture) requires the development of computational methods and models of increased accuracy and adequacy and their implementation in modern computer-aided design systems. The solution to this problem is associated with the need for tests that go beyond standard uniaxial tension and compression tests in order to obtain more complete information about the mechanical response of materials to physical and mechanical influences that occur during the manufacturing and operation of structures.

Forging technologies in a wide range of strain rates, including promising technologies such as electromagnetic forming, electrohydraulic forming, explosive forming, impact hydroforming, etc., are used in the manufacture of structural elements from sheets of alpha and near alpha titanium alloys for aerospace, aircraft engines, power equipment, and chemical technological equipment.

Alpha titanium alloy Ti-5Al-2.5Sn (Grade 6) is employed both at low and cryogenic temperatures and at elevated temperatures up to 753 K [1,2]. It should be noted that the Ti-5Al-2.5Sn alloy has high fracture toughness in cryogenic media and has been successfully used to create containers for storing hydrogen at cryogenic temperatures. This increases the relevance of investigating the possibilities to prevent the occurrence of microdamage in the manufacturing process of Ti-5Al-2.5Sn thin-walled hydrogen tanks and vessels. The Ti-5Al-2.5Sn alloy has good oxidation resistance and good weldability but is poorly amenable to machining by cutting [3]. Therefore, the production of thin-walled structural elements from the Ti-5Al-2.5Sn alloy is currently performed by quasi-static forging and forging in the superplasticity mode [4,5]. One of the promising technological directions for the production of thin-walled structural elements is dynamic stamping [6,7].

The mode of the stamping process, the accuracy, and the quality of the resulting structural elements are influenced by a number of factors, including the strain rate, the distribution of the stress state and plastic deformation over the thickness of the alloy, the initiation of damage, and the formation of micro- and macrocracks. It should be also noted that the results of studies of the general regularities of deformation and fracturing of alpha-titanium alloys with a hcp crystal lattice, obtained during punching tests, can be used to validate constitutive relationships, damage, and fracture models for other hcp alloys based on Zr, Be, Hf, etc. [8]. The punching test of thin plates of metals and alloys with hemispherical indenters, including those of small diameter, is one of the tests that allows studying of the mechanical behavior of materials during elastic–plastic deformation under conditions of a complex stress state [9]. Quasi-static and dynamic punch tests with hemispherical or small indenters are performed in accordance with accepted standards on thin specimens of metals and alloys (ASTM E3205, the European standard BS EN 10371, CWA 15627, GOST 10510-80, the Chinese standard GB/T 29459), polymeric (ASTM F2977) and composite materials, and materials obtained by additive technologies (ASTM WK61832) [9,10,11,12,13,14,15,16,17].

The results of punch tests can be used for additional verification of models of the mechanical behavior of materials developed on the basis of data obtained in experiments under uniaxial loading [9,10,11,12,13,14,15,16,17]. The results of punch tests in a wide range of loading rates can be used to create methods for modeling dynamic punching processes and predicting punching modes, springback effects, distribution of plastic deformations, and damage initiation.

Numerical modeling of the processes of deformation, nucleation, and growth of damage and fracture of sheets of titanium alloys during penetration at a wide range of punch speeds makes it possible to calibrate models of the mechanical behavior of the materials as well as to modify them in order to increase the accuracy of theoretical predictions when designing technological modes for pressing thin sheets of alpha titanium alloys [17,18,19]. Characteristics of the mechanical properties of titanium alloys, including the values of yield strength, tensile and compressive strength, strain hardening characteristics, and crack resistance, are used in computer designs of light and reliable structures. Note that today, there is no universal approach for determining such characteristics for materials in a wide range of strain rates, temperatures, the stress triaxiality parameter, or the Lode parameter [8,20,21,22,23].

In a number of recent works, the mechanical behavior of titanium alloys in a wide range of strain rates has been studied by an inverse modeling method based on a numerical analysis of results of tests for penetration of a spherical body through flat samples [24,25,26,27,28,29]. The results obtained during punch tests of sheet metals and alloys at relatively low punch speeds are widely used to validate computational models and to determine the mechanical properties of materials under quasi-static deformation. Hähner et al., Abendroth et al., and Lancaster et al. used results of quasi-static punch tests to determine tensile mechanical properties of fcc, bcc, and hcp metals and alloys and to verify constitutive relations for steels and nickel alloys [27,28,29]. Huang et al., as well as Safari et al., showed the possibility to use the results of quasi-static punch tests to determine the tensile mechanical properties of fcc, bcc, and hcp metals and alloys as well as to determine the metal forming limits in stamping [30,31]. The quasi-static punch tests are also used for determination of the ductile-to-brittle transition temperature [32]. 

Sun et al., and Zhao et al. proposed methods for determining the parameters of constitutive equations and the GTN (Gurson–Tvergaard–Needleman) damage model based on the analysis of test results of the quasi-static punching of sheet metals and alloys [33,34]. It was shown that the test scheme is highly informative, and the experimental results obtained with its application can be used to verify the constitutive equations and models of fracturing of metals and alloys.

The aim of this work was to study the mechanical behavior of titanium alloy Ti-5Al-2.5Sn at high strain rates, which are realized when a thin plate is penetrated by a hemispherical punch at a velocity of 0.3 mm/s to 15 m/s. The obtained data supplement the results of earlier studies of the mechanical behavior of titanium alloys under dynamic loading with the variation of the stress triaxiality parameter [19,20,21,22].

## 2. Materials and Method of Punching Test

### 2.1. Material and Experimental Setup

The studies were carried out on commercial thin-sheet rolled products of Ti–5Al–2.5Sn (analogous to alloy VT5). Alloy VT5-1 had a chemical composition (wt. %) of: 5.311 Al, 2.51 Sn, 0.211 Fe, 0.0116 Si, 0.864 V, and 0.2786 Zr, Ti—balance.

**Figure 1:**
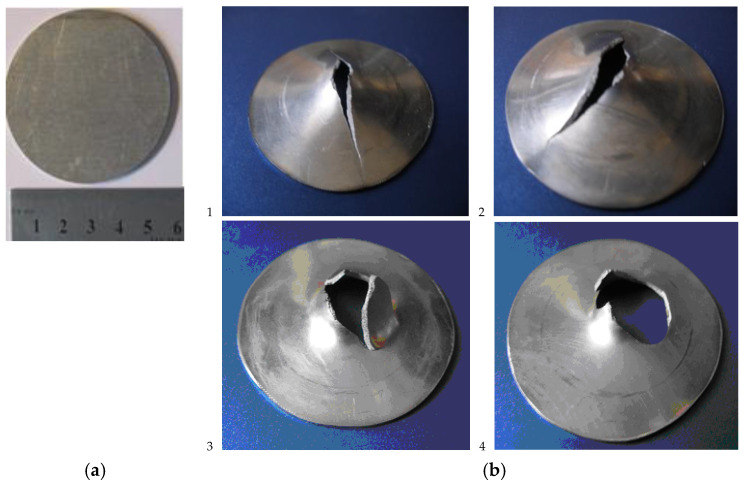
(**a**) Schematic geometry of specimen; (**b**) Photos of specimens after testing at the velocities (**b1**) 0.0003 m/s, (**b2**) 0.003 m/s, (**b3**) 2 m/s, and (**b4**)—15 m/s.

The alloy was in the polycrystalline state. The grain structure of the alloy was studied on a TESCAN scanning microscope by secondary electron diffraction (EBSD). The obtained scans were analyzed using licensed software HKL Channel 5 Version 5.12.74.0 Oxford Instruments (Abingdon, UK). The structure was fine-crystalline, homogeneous, and equiaxed. The average grain size of the alloy was ~40 μm. The test specimens were cut by the electric spark method on a DK7732 CNC machine from a sheet 1.2 ± 0.05 mm thick, which ensured high accuracy in reproducing the geometric parameters of the specimens. The surfaces of the samples were polished to reduce the friction coefficient in contact with the punch. Geometry parameters of samples for high-speed testing were selected in accordance with INSTRON recommendations and ASTM E643-09 and ISO 8490-86 standards. Figure 1a shows the geometry of the specimen and photos of specimens after testing. The diameter of specimen *d* is 60 ± 0.1 mm. The diameter in the lower part of the support matrix is 42 ± 0.1 mm.

Tests at a constant punching velocity were carried out by an Instron VHS 40/50-20 high-speed test bench (Instron, High Wycombe, UK) with a 50 kN load cell. Tensile forces and displacements were recorded up to fracture of specimen with a high temporal resolution. The load was recorded using the certified dynamic force sensor Kistler. The sensor was calibrated for a load of 50 kN, allowing a maximum load of 60 kN and a maximum overload of 72 kN. The load measurement accuracy is 0.25% in the range from 1% to 100% of the rated load of the sensor. The load ringing phenomenon occurs in high-velocity tests due to the relatively low eigen frequency of the load sensor. Therefore, the load–time curves recorded on the Instron machine underwent filtering to reduce the high-frequency signals [24].

**Figure 2:**
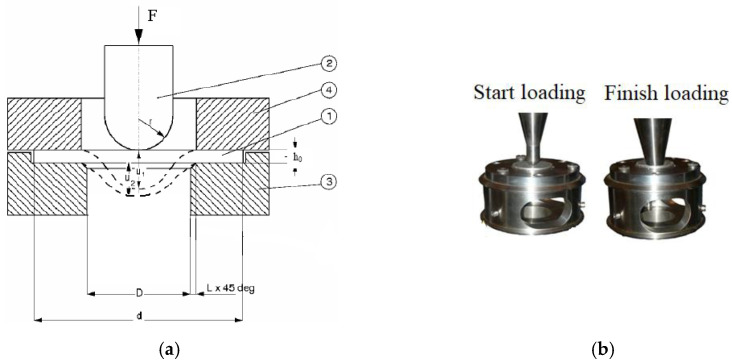
(**a**) The sectional scheme of punch test setup: 1—specimen; 2—hemispherical punch; 3, 4 are the lower and the upper parts of the support matrix, respectively; (**b**) Photos of punch positions at the start and end of the punching test process at Instron VHS 40/50-20 servo-hydraulic bench.

The force sensor was connected to the punch, and the sample fixed in the supporting matrix moved at a given constant speed in the direction of the punch. The tests were conducted in the speed control mode at the initial values of longitudinal velocities: 0.0003 ± 0.00001, 0.003 ± 0.0001, 2 ± 0.01, and 15 ± 0.1 m/s. The application of a servo-hydraulic drive in the VHS 40/50-20 system eliminates the influence of gaps between the elements of the loading system on the loading modes, which takes place when using universal screw testing machines. Figure 2a shows the sectional scheme of punch test setup. Figure 2b shows the positions of punch positions at the start and end of the punching test process.

Note that the punching speed affects the dynamics of plate failure and the nature of the formation of cracks. The change in the nature of fractures with an increase in the punching rate is due to the development of localization of plastic deformation in the zone of intense tension. 

**Figure 3:**
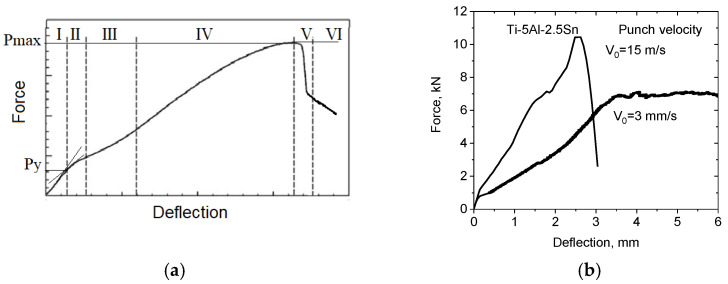
(**a**) Diagram of punching force versus the maximum displacement of the specimen; (**b**) Experimental F(*u*_z_) diagram for Ti-5Al-2.5Sn.

The velocity of punching the specimen was determined during the tests by integrating the data obtained from the acceleration sensor using the HV 8800 Version 7 software. Data on displacement of the central point of the specimen were determined in the HV 8800 software from the difference in data of the position sensor. A typical diagram of the punching compression force as a function of the maximum sample displacement in the punching direction is shown in Figure 3.

Six stages can be distinguished on the F(*u*_z_) diagram [35,36].

The first (I) stage corresponds to the elastic deformation of the plate under punching. The second (II) stage corresponds to the violation of linearity. The third (III) stage corresponds to the initiation of plastic deformations. The fourth (IV) stage corresponds to the development of plastic deformations in the sample under punch movement. At the fifth (V) stage, a decrease in the deformation force is recorded as a result of the formation of cracks and the formation of a fracture zone of the sample, and the sixth (VI) stage corresponds to the partial fragmentation of the sample at the final stage of fracture.

The true macroscopic stress σ*_s_* can be determined by the relation [10,35,36]:

(1) $$\sigma_{s}=\alpha \;P_{y}/h^{2}$$

where α is the parameter of material and *P_y_* is the load value at the beginning of plastic flow.

The value of *P_y_* load is determined in the transition between zones I and II of the curve. *P_y_* load can be determined by finding the point of intersection of the punching diagram with a straight line parallel to stage 1 and displaced by 0.1 *h* or 0.01 *h*, where *h* is the thickness of the specimen [37].

The strongest correlation with yield strength σ*_y_* can be described by relation [10,35]:

σ*_y_* = α_1_ *P_y_*/*h*^2^ + α_2_, (2)

where α_1_ and α_2_ are test constants.

The value of *P_y_* in (2) was proposed to be determined as the crossing point of two tangents defined in the elastic regime (zone I) and the plastic regime (zone II) [20]. Modification of method, in which *P_y_* is defined as the vertical projection of the crossing point of the two tangents on the test curve, was also proposed [38].

The maximum punch load *P_max_* indicated in a F(*u*_z_) curve has been shown to exhibit a linear correlation with the ultimate tensile strength (σ*_UTS_*).

Σ*_UTS_* = β_1_ *P_max_*/*h*^2^ + β_2_, (3)

where β_1_ and β_2_ are constants of material and the coefficient of friction, and *h* is the initial sample thickness.

When displacements are at maximum load, *h_f_* is the thickness of the sample at the edge of the crack, associated with the elongation of the material in tension.

Strain to fracture ε*f* can be defined from the experimental measurement of the final thickness of the specimen in the fracture region by relation (4) [35,36]:

(4) $$\varepsilon_{f}\approx \ln(h_{0}/h_{f})$$

(5) $$\varepsilon_{f}\approx \gamma (d_{max}/h)$$

where γ is the material parameter, *h_f_* is the sample thickness at the fracture location of the specimen, *h*_0_ is the initial sample thickness, and *d_max_* is the sample deflection at the point of *P_max_*.

### 2.2. Numerical Simulation of Plastic Flow and Damage Evolution of Titanium Alloy during Dynamic Punching Test

The computational model utilizes the theoretical basis of continuum damage mechanics. The dynamics of the deformation process were described by the system of equations that included equations of conservation of mass, momentum, and energy (5), kinematic relations (6), constitutive relations (7) in the form of an equation of state (8), and a relaxation equation for the deviatoric stress tensor (9) [8]:

(6) $$\frac{d\rho }{dt}=\rho \frac{\partial u_{i}}{\partial x_{i}},\;\frac{\partial \sigma_{ij}}{\partial x_{j}}=\rho \frac{du_{i}}{dt},\;\rho \frac{dE}{dt}=\sigma_{ij}{\dot{\varepsilon }}_{ij}.$$

(7) $${\dot{\varepsilon }}_{ij}=(1/2)[\partial u_{i}/\partial x_{j}+\partial u_{j}/\partial x_{i}),\;{\dot{\omega }}_{ij}=(1/2)[\partial u_{i}/\partial x_{j}-\partial u_{j}/\partial x_{i}).$$

(8) $$\sigma_{ij}={\sigma_{ij}}^{\left(m\right)}\varphi (f),\;\sigma_{ij}^{\left(m\right)}=-p^{\left(m\right)}\delta_{ij}+{S_{ij}}^{\left(m\right)},$$

(9) $$\begin{array}{c} p^{\left(m\right)}=p_{{}_{x}}^{\left(m\right)}(\rho )+\Gamma (\rho )\rho E_{T},\;E_{T}=C_{p}T,\\ {p_{x}}^{\left(m\right)}=\frac{3}{2}B_{0}\cdot ({\left(\rho_{0}/\rho \right)}^{-7/3}-{\left(\rho_{0}/\rho \right)}^{-5/3})[1-\frac{3}{4}(4-B_{1})\cdot ({\left(\rho_{0}/\rho \right)}^{-2/3}-1)] \end{array}$$

(10) $$\begin{array}{c} DS_{ij}^{\left(m\right)}/Dt=2\mu ({\dot{\varepsilon }}_{ij}^{e}-\delta_{ij}{\dot{\varepsilon }}_{kk}^{e}/3),\\ {\dot{\varepsilon }}_{ij}^{}={\dot{\varepsilon }}_{ij}^{e}+{\dot{\varepsilon }}_{ij}^{p},{\dot{\varepsilon }}_{ij}^{p}={\dot{e}}_{ij}^{p}+\delta_{ij}{\dot{\varepsilon }}_{kk}^{p}/3,\;{\dot{e}}_{ij}^{p}=\lambda \partial \Phi /\partial \sigma_{ij},\;{\dot{\varepsilon }}_{kk}^{p}={\dot{f}}_{growth}/(1-f) \end{array}$$

where *ρ* is the mass density, *u_i_* is the component of the particle velocity vector, xi are coordinates, E is the specific internal energy, ${\dot{\varepsilon }}_{ij},\;{\dot{\omega }}_{ij}$ are the components of strain rate tensor and the bending–torsion tensor, *φ*(*f*) is a function relating the effective stresses of the damaged medium to the stresses in its condensed phase, ξ = *ρ*^(m)^/*ρ*^(m)^_0_, Γ = (γ_R_ − 0.5) (*ρ_R_*/*ρ*)*n* − 0.5 is the Grüneisen coefficient, *ρ*^(*m*)^_0_ is the initial mass density of the condensed phase of the alloy, *γ_R_*, *ρ_R_*, *n*, *B*_0_, and *B*_1_ are the material parameters, *C_p_* is the specific heat capacity *D*(.)/*Dt* is the Jaumann derivative, *μ* is the shear modulus, *f* is the void volume fraction, $\dot{\lambda }$ is the plastic multiplier derived from the consistency condition $\dot{\Phi }=0$, Φ is the plastic potential in Gurson–Tvergaard–Needleman (GTN) model, and ${\dot{f}}_{growth}$ is the void growth rate. The function *φ*(*f*) takes the form (1 − *f*) for pressure and is implicitly defined by the Gurson–Tvergaard equation for the deviatoric stress tensor [20,39].

The temperature increase due to dissipation of energy during plastic flow is calculated by relation [12]:

(11) $$T=T_{0}+\int_{0}^{\varepsilon_{eq}^{p}}(\beta /\rho \;C_{p})\;\sigma_{eq}d\varepsilon_{eq}^{p},$$

where *T*_0_ is the initial temperature, β~0.9 is the parameter representing the fraction of plastic work converted into heat, and σ*_eq_* is the equivalent stress.

The temperature dependence of the specific heat capacity for Ti-5Al-2.5Sn titanium was obtained by the phenomenological relations within temperature *T* range from 293 to 1115 K [40].

(12) $$C_{p}=248.389+1.53067T-0.00245T^{2}\;[J/kgK]\;at\;0<T<T_{\alpha \beta }=1320\;K$$

The temperature dependence of the shear modulus for the alpha titanium alloy was calculated by the equation [41]:

(13) $$\mu (T,\;\rho )=\mu_{0}[1+\mu_{1}\;p(\rho_{0}/\rho )-\mu_{2}(T-295)]$$

The yield stress of condensed phases was described using following constitutive relation [8,23]:

(14) $$\sigma_{s}^{disl}=(C_{0}{\left(\varepsilon_{eq}^{p}\right)}^{1/2}+k_{hp}^{disl}{d_{g}}^{-1/2}\;)\exp(-A_{0}T)\exp(A_{1}T\ln(1+\frac{{\dot{\varepsilon }}^{p}{}_{eq}}{{\dot{\varepsilon }}^{p}{}_{disl\;0}})),$$

where $k_{hp}^{disl}$, *C*_0_, *A*_0_, and *A*_1_ are material parameters, *d_g_* is the grain size, ${\dot{\varepsilon }}_{0}^{p}$ is parameter normalizing the plastic strain rate, and $\varepsilon_{eq}^{p}$ is the equivalent plastic strain,

The material coefficients of Ti-5Al-2.5Sn are shown in Table 1.

Because plastic deformation and damage develop non-homogeneously and non-stationary in the punch test of specimen, it is difficult to ensure the accuracy of predictions on the evolution of damage during the plastic deformation by analytical formulas or 1D simulations. In this paper, a 3D numerical simulation technique was used to quantitatively analyse the influence of the triaxial stress state on the void nucleation, growth, and coalescence in the titanium alloy under tension in biaxial stress-loading conditions. The GTN model was employed to describe the influence of damage on the flow stress [8,20,39]:

(15) $$(\sigma_{eq}{}^{2}/\;\sigma_{s}{}^{2})+2q_{1}f^{*}\;\cosh(-q_{2}\;p/2\sigma_{s})-1-q_{3}{\left(f^{*}\right)}^{2}=0$$

where σ*_s_* is the yield stress, *p* is the pressure, *q*_1_, *q*_2_, and *q*_3_ are model parameters, and *f* is the void volume fraction. 

The Needleman void size evolution model was applied to describe the kinetics of damage during the plastic deformation [8,13,23,28]. The void growth rate depends on the dilatant part of the plastic strain. Therefore, no void growth is assumed in pure shear deformation of the isotropic material.

The void nucleation depends on the equivalent plastic strain ɛ_p_ and assumes a normal size distribution of nucleated voids [13,16,17,28].

(16) $$\begin{array}{l} \dot{f}={\dot{f}}_{nucl}+{\dot{f}}_{growth},\\ {\dot{f}}_{nucl}=[f_{N}/(\sqrt{2\pi }\;s_{N})]\;\exp\;\{-0.5{\left[({\varepsilon^{p}}_{eq}-\varepsilon_{N})/s_{N}\right]}^{2}\}{\dot{\varepsilon }}_{eq}^{p},\\ {\dot{f}}_{growth}=(1-f){\dot{\varepsilon }}_{kk}^{p}, \end{array}$$

where ε*_N_* and *s_N_* are the average nucleation strain and the standard deviation, respectively. The volumetric concentration of nucleating voids in the condense phase is controlled by the parameter *f_N_*, ${\dot{\varepsilon }}_{eq}^{p}={\left[(2/3){\dot{\varepsilon }}_{ij}^{p}{\dot{\varepsilon }}_{ij}^{p}\right]}^{1/2}$.

(17) $$\begin{array}{l} f^{*}=f\;if\;f\leq f_{c};\\ f^{*}=f_{c}+({\bar{f}}_{F}-f_{c})/(f_{F}-f_{c})\;if\;f>f_{c}, \end{array}$$

where ${\bar{f}}_{F}=(q_{1}+\sqrt{q_{1}{}^{2}-q_{3}})/q_{3},$ *q*_1_, *q*_2_, and *q*_3_ are constants of the model.

The final stage of damage evolution prior to ductile fracture corresponds to the voids’ coalescence into the macroscopic crack. The linkage of voids during coalescence causes softening of the material and an accelerated growth rate of the void fraction f* up to the element erosion condition (when void fraction *f_F_* reaches its critical value).

**Figure 4:**
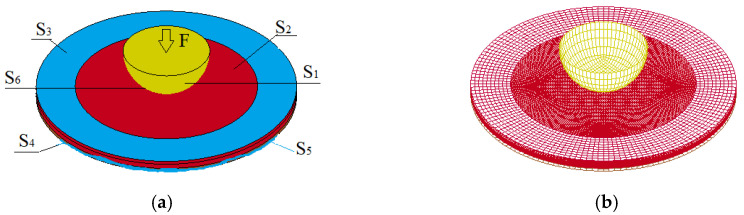
(**a**) Numbering of surfaces in the computational model; (**b**) Cells of the specimen and punch.

The GTN model of ductile fracture requires assignment of nine parameters: three Tvergaard’s parameters of yield surface (*q*_1_, *q*_2_, and *q*_3_), the initial void fraction f_0_, three parameters of nucleated void size distribution (ε_N_, s_N_, and f_N_), and two parameters determining the kinetics of void coalescence (*f_c_* and *f_F_*). The model parameters for the Ti-5Al-2.5Sn titanium alloy were obtained throughout the semi-inverse numerical simulation technique of uniaxial tension tests at the strain rates 0.1, 100, and 1000 s^−1^ [20,23]. The algorithm for determining the GTN model parameters using combined numerical and experimental technique can be found in [42,43]. The model has been applied to simulate punching of thin titanium plates by a hemispherical indenter. The following values of the model coefficients for Ti-5Al-2.5Sn were used in the calculations: *q*_1_ = 1.0, *q*_2_ = 0.7, *q*_3_ = 1.0, *f*_0_ = 0.003, *f*_N_ = 0.1156, *f*c = 0.117, *f*_F_ = 0.260, ε_N_ = 0.25, and *s*_N_ = 0.05. The finite element model assumes that the material is isotropic with an elastic–viscoplastic response. The discretization of computational domains were done by eight-node linear hexahedral solid elements with reduced integration and hourglass control. Because the spatial step size may influence the energy dissipation process associated with damage evolution, a fine mesh with an edge length of 0.3 mm was introduced. The model of the titanium plate utilized ~200,000 elements. Figure 4a shows a numbering of surfaces in the computational model. Figure 4b shows the grid models of the sample and punch.

The loading velocity is applied on the rigid body indenter, which is consistent with the punching test. Constraint of displacement is applied on the one side of specimen.

Boundary conditions corresponding to constant loading rate have the form:

(18) $$u_{3}{|}_{S_{1}}=V_{0},\;i=1,2,3\;u_{i}{|}_{S_{3}\cup S_{4}}=0,\;i=1,2,3;\;\sigma_{ij}{|}_{S_{5}}=0,\sigma_{ij}{|}_{S_{2}}\cap {}_{S_{6}}=0,\;u_{i}{}^{+}{|}_{S_{6}}=u_{i}{}^{-}{|}_{S_{6}},\;i=1,\;2,\;3;\;p^{+}{|}_{S_{6}}=-p^{-}{|}_{S_{6}},\;i=1,\;2,\;3;\;\sigma_{ij}{}^{+}|{\;}_{S_{6}}=\sigma_{ij}{}^{-}|{\;}_{S_{6}}=0,\;i\neq j.$$

where $u_{i}{|}_{S_{j}}$ are the components of the particle velocity vector on the surface *Sk*, k = 1…6, σ*_ij_* represents the components of the stress tensor, *p* is the pressure, and *V*_0_ is the velocity of rigid punch.

The initial conditions corresponded to the relaxed stress state and uniform temperature field.

The numerical simulations were carried out on the LS DYNA solver (ANSYS WB 15.2 software). The constitutive relations were implemented in the explicit solver by writing a UMAT subroutine. The implicit return mapping algorithm for the Gurson-type yield potential and elastic–viscoplastic medium can be found in [44]. The calculations were done using a finite-difference scheme of second order accuracy. 

## 3. Results

### 3.1. Experimantal Results

**Figure 5:**
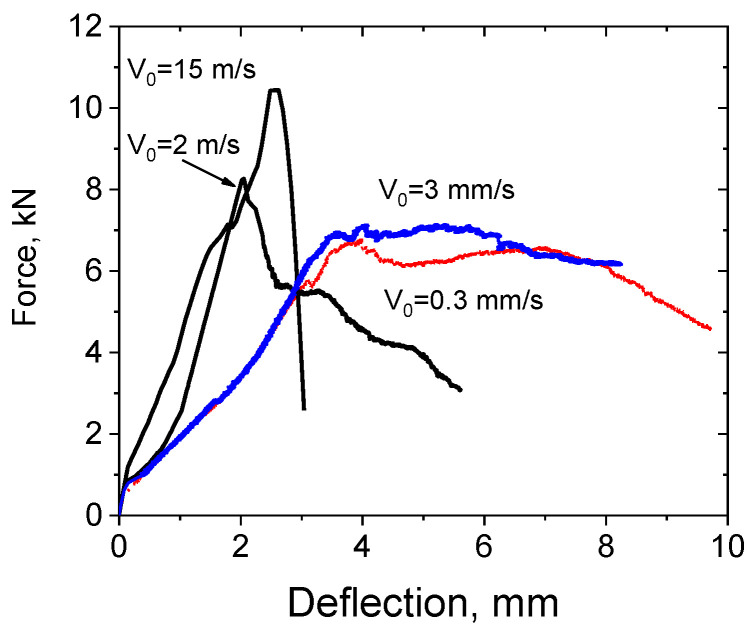
Experimental F(*u*_z_) diagrams for Ti-5Al-2.5Sn.

Titanium plates were tested at punch speeds of 0.0003, 0.003, 2, and 15 m/s on an Instron VHS 40/50-20 servo-hydraulic bench. Experimental F(*u*_z_) diagrams are shown in Figure 5.

The resultant F(*u*_z_) force diagrams of resistance to penetration of a hemispherical punch from its movement indicate ductile fracture at punch speeds up to 15 m/s. The results indicate no embrittlement of the Ti-5Al-2.5Sn alpha titanium alloy under high-speed punching conditions. The formation of cracks was preceded by significant plastic deformation. Note that the general behavior of the punching force versus deflection is preserved, as shown in Figure 3a.

The *P_y_* was determined by the bilinear method [35,36]. Using these values, the uniaxial tensile yield stress was evaluated from relationship (2). The coefficient α_1_ was estimated as 1.489. The use of IPF ER-3 grade lubricant between the punch and the sample surface reduced the friction during punching. As a result, the value of the coefficient α_2_ was reduced. The yield strengths of the Ti-5Al-2.5Sn alpha titanium alloy were estimated to be 1.2 GPa at a die speed of 15 m/s and 0.83 GPa at indenter velocities of 3 mm/s and 0.3 mm/s.

The ultimate tensile strength (σ*_UTS_*) of Ti-5Al-2.5Sn during dynamic punching was estimated by the relation (3). The maximum punch load *P*_max_ on the *F*(u_z_) curve depends on the punch speed, as shown in Figure 5. The maximum forces *P*_max_ in the experiments on quasi-static punching of titanium plates were close and lie in the confidence range for three tests for each punch speed. The ultimate tensile strength values varied from 1.285 ± 0.04 GPa to 1.01 ± 0.03 GPa and 0.862 ± 0.03, 0.860 ± 0.03 GPa at punch speeds of 15 m/s, 2 m/s, 3 mm/s, and 0.3 mm/s, respectively. 

Dynamic punching tests made it possible to evaluate the increase in the resistance to fracture of alloys under high-speed non-uniaxial tension. Note that the values of the coefficients α_1_ and β_1_ in relations (2) and (3) depend on the stress triaxiality that is realized during the deformation of the plate. The strain and stress states of the plates before failure are affected not only by the thickness of the samples, but also by the shape and dimensions of the punch. Therefore, the use of specific values of the coefficients α_1_, β_1_, α_2_, and β_2_ in relations (2) and (3) is justified for specific test devices. The values of the coefficients α_2_ and β_2_ depend on the friction between the surfaces of the punch and the specimen plate. The values of α_2_ and β_2_ can be reduced by polishing both surfaces and by applying a lubricant based on molybdenum disulphite between the surfaces.

The strains to failure were calculated by Formula (4). The thicknesses of the plates were measured at the boundary of the fracture zone. Note that in the case of a non-circular crack or non-symmetrical cracks in the samples, as shown in Figure 1b, the values of the strain-to-fracture ε*_f_* can vary significantly in different parts of the crack boundary. The obtained values of ε*_f_* were equal to 0.25 ± 0.02, 0.35 ± 0.02, and 0.42 ± 0.02 at the punch velocities of 15, 2 m/s, and 3 mm/s, respectively. These values correlate with the experimental fracture strains of Ti-5Al-2.5Sn subjected to uniaxial tension of notched specimens at strain rates from 10^3^ to 0.1 s^−1^ [20].

The obtained values of the strain-to-fracture can be used for comparisons with the results of numerical simulation of the deformation process including the development of damage and the formation of cracks during the punching.

### 3.2. Simulation Results

**Figure 6:**
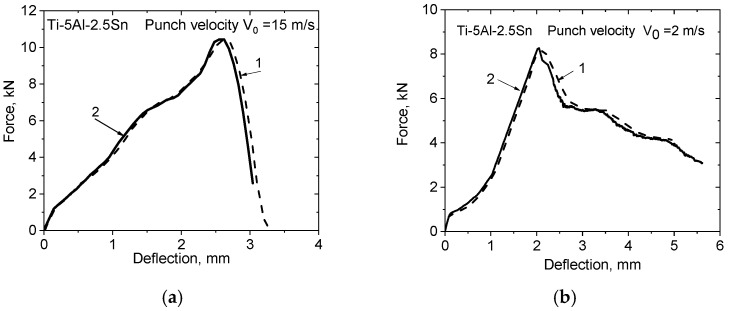
(**a**) Diagrams F(*u*_z_) for Ti-5Al-2.5Sn at an indenter speed of 15 m/s; (**b**) Diagram F(*u*_z_) at a stamp speed of 2 m/s. Curves 1 were obtained by numerical simulation, curves 2 were obtained experimentally.

A numerical simulation of the punching process of sheets of Ti-5Al-2.5Sn titanium alloy in the range of punch speeds from 0.3 mm/s to 15 m/s was used to study the regularities of the processes of deformation, damage, and fracture. The results of the calculations confirmed the possibility of using a computational model that includes Equations (6)–(17) and boundary conditions in the form (18) in the numerical simulation of high-speed punching of thin titanium plates by a hemispherical punch. The calculated dependences of the punching force on the displacement of the central point of the contact zone between the plate and the indenter at punching speeds of 15 and 2 m/s are shown in Figure 6. Line 1 denotes the calculation results, whereas line 2 denotes the experimental data.

**Figure 7:**
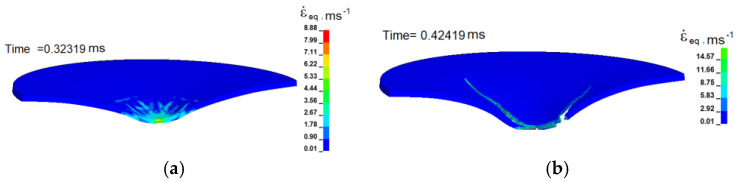
(**a**) Calculated distribution of effective strain rates in the cross section of punched Ti-5Al-2.5Sn plate at the time of 0.32319 ms and (**b**) at the time of 0.42419 ms. The velocity of the punch is 15 m/s.

Calculated and experimental force versus deflection curves have good correlation at the dynamic punching. The calculated distribution of effective strain rates in the cross-section of a punched Ti-5Al-2.5Sn plate at the 15 m/s velocity are shown in Figure 7. The strain rate in the plastic drawing zone can vary within range from 100 to 4000 s^−1^. The strain rate in the local zone near the crack formation can be higher than these values.

**Figure 8:**
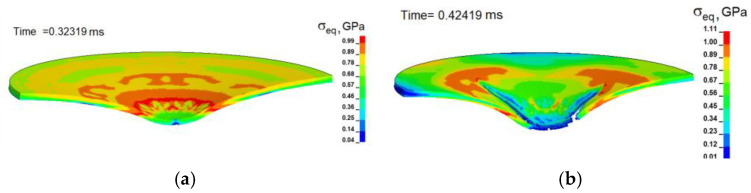
(**a**) Calculated distribution of equivalent stress in the cross-section of a punched Ti-5Al-2.5Sn plate at the time of 0.32319 ms and (**b**) at the time of 0.42419 ms. The velocity of the punch is 15 m/s.

The simulation results showed that the plastic deformation of the titanium alloy develops non-homogeneously in the punching zone of the sample both along the thickness of the sheet and along the contact surface. The localization of plastic deformations is associated with a non-uniform distribution of equivalent stresses in the deformation zone, as can be seen in Figure 8. The values of equivalent stresses obtained in the calculation are consistent with the experimental values of the flow stress of this titanium alloy in the corresponding range of strain rates and the values of stress triaxiality [13].

**Figure 9:**
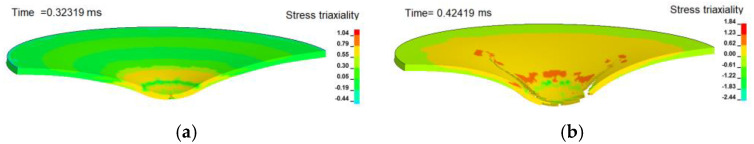
(**a**) Calculated distribution of stress triaxiality parameter in the cross-section of a punched Ti-5Al-2.5Sn plate at the time of 0.32319 ms and (**b**) at the time of 0.42419 ms. The velocity of the punch is 15 m/s.

Thus, the high enough equivalent stresses can create in thin plates at relatively small deflections during dynamic punching. The calculated values of the stress triaxiality parameter η = −σ_eq_/p in the section of the deformable plate are shown in Figure 9. 

The simulation results showed that the deformation of the material in the punching zone proceeds with a non-stationary and spatially inhomogeneous change in the stress triaxiality parameter. Improving the accuracy of the CAE analysis of high-speed stamping of the titanium sheet requires a material model that takes into account the effect of stress triaxiality. 

**Figure 10:**
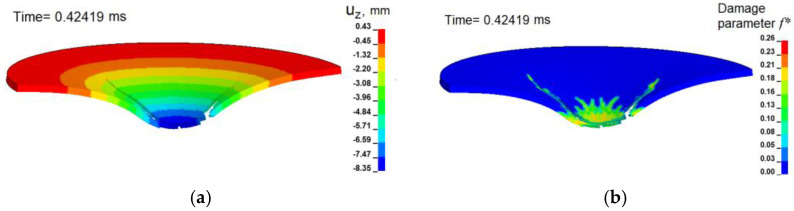
(**a**) Calculated distribution of displacements u_z_ in the cross-section of a punched Ti-5Al-2.5Sn plate at the time of 0.42419 ms; (**b**) is the distribution of the damage parameter in the cross-section of a punched plate. The velocity of the punch is 15 m/s.

The distributions of the calculated displacements and the damage parameter in the section of the deformable sample during the opening of the formed cracks are shown in Figure 10. The distributions are typical for stage V of the diagram of punching force versus the maximum deflection of the specimen (See Figure 3a).

Asymmetric cracks shown in Figure 1b that appear in plates when exposed to a hemispherical punch with axial symmetry are a consequence of the localization of high-speed plastic deformation that takes place in metals with a hexagonal close-packed crystal lattice, which include the alloy studied in this work. 

The method of testing metallic materials during punching made it possible to obtain the average characteristics of the yield strength, ultimate strength, and ultimate strain to failure and to determine the regularities of nucleation and growth of micro-damage. Verification of the damage kinetics requires detailed data on the development of damage at the fifth stage and the sixth stage of the punching process.

## 4. Discussion

The research results in this work showed that the spatial distribution of damage and crack shapes in a thin sheet of titanium alloy during stamping are significantly affected by the speed of the punch. The change in the geometry of cracks in the range of punch speeds from 0.0003 to 15 m/s can be seen in the photograph of the samples after testing in Figure 1b. Mayer and Pursche [45] noted that the samples actually exhibit a biaxial stress–strain state when testing for punching. There are three types of failure under the specified loading conditions: normal, radial, or ring failure. Radial cracks form under conditions of low plasticity of the material. A combination of radial cracks and bands of radial-circumferential shear are observed at high-stressed states. Many annular cracks and many bands of radial and circumferential shear are formed at low plasticity of the material. The observed changes in the geometric parameters of the resulting cracks are consistent with the data obtained on the increase in the flow stress and tensile strength with an increase in punch speed.

Thus, qualitative agreement between the distribution and geometric shapes of formed cracks in the calculations and the characteristics observed in experiments confirms the adequacy of the computational model and the capability of the prediction of fracture of alpha titanium alloys at high strain rates in a biaxial stress–strain states.

An increase in the strain rate under conditions of biaxial stress–strain state promotes the formation of localized shear bands. The formation of localization bands is related to the instability of the plastic flow process. Equation (14) can be used for prediction of the plastic flow instability, as shown in [8,21]. Equation (14) involves the description of the strain hardening, strain rate sensitivity, and temperature sensitivity of the yield stress of the Ti-5Al-2.5Sn alloy within the condensed phase of undamaged material.

Due to the relatively low values of the thermal conductivity coefficient for alpha titanium alloys, the nucleation of localization bands and the formation of zones of localization of plastic deformation during the initiation of damage is accompanied by local heating of the material. The effect of heating in the zones of damage formation and fracture of titanium alloys was previously observed by the infrared thermometry [46]. Roessig and Mason [47] as well as Bai and Dodd [48] studied the adiabatic shear band formation in various media, including the titanium alloys. The results of the studies showed that adiabatic shear bands are formed in titanium alloys under high-velocity impact of a punch. Localized adiabatic shear bands are readily formed in titanium alloys due to the relatively high strength and low strain hardening of the alloys. Thus, the probability of localized shear formation in the titanium alloy during punching increases with increasing punch velocity. As a result, circumferential bands of strain localization are formed in the plate. A crack is formed in this zone due to damage nucleation and growth. A similar circumferential half-crack was formed in the sample after punching with a velocity of 15 m/s (see Figure 1(b4)).

We used a GTN damaged medium model including Equations (16) and (17) to describe the process of crack formation during dynamic punching. The results of numerical simulations of the fifth and sixth stages of punching agree satisfactorily with the experimental results, as shown in Figure 6. The results obtained in this work are consistent with the findings of recent studies [28,49]. Martínez-Pañeda et al. achieved good agreement in describing the fifth and sixth stages of the punching process in chromium steel using the GTN model [49]. Lancaster et al. showed the possibility of an adequate description of the fracture of the plates of alloy Ti-6Al-4V during punching at a low speed.

## 5. Conclusions

The mechanical behavior of Ti-5Al-2.5Sn has been investigated using the punch test. The punch test was performed utilizing the semispherical punch of 20 mm diameter at the velocities of 0.0003, 0.003, 2, and 15 m/s. The tests were carried out using the Instron VHS 40/50-20 servo-hydraulic bench.

The results can be summarized as follows:

1. The maximum values of plastic strain in the crack zone of fractured plates significantly exceeded the values of the macroscopic elongation δ of Ti-5Al-2.5Sn available in literature.
2. The high local equivalent strains can realize in thin titanium plates at relatively small deflections during dynamic punching.
3. The spatial distribution of damage and crack shape in a thin sheet of titanium alloy during stamping is significantly affected by the speed of the punch.
4. An increase in the strain rate under conditions of a biaxial stress–strain state promotes the formation of localized shear bands and damage nucleation.
5. The stress triaxiality parameter in the sheet of titanium alloy changes non-stationary and spatially inhomogeneous in the punching zone under deformation and damage development.

The used computational model proposed in [8] makes it possible to describe regularities of plastic flow and fracture of the Ti-5Al-2.5Sn alloy in a wide range of strain rates and under biaxial stress–strain state.

The obtained experimental data on the mechanical behavior of the Ti-5Al-2.5Sn alloy in a wide range of strain rates and in a biaxial stress state can be used in the design of thin-sheet titanium products and the technology of its manufacture by stamping.

## Figures and Tables

**Table 1:** Material coefficients.

Material	Parameter					
Ti-5Al-2.5Sn	*C*_0_, GPa	$k_{hp}^{disl}$, GPa μm^1/2^	*A*_0_, K^−1^	*A*_1_, K^−1^	${\dot{\varepsilon }}^{p}{}_{disl\;0}$, s^−1^	*T*_m_, K
	0.665	0.628	0.0224	0.002	10^3^	1875

## Data Availability

Data obtained within this research and supporting reported results are referenced in the report on grant No. 22-79-00162.
